# Metabolic responses to ethanol and butanol in *Chlamydomonas reinhardtii*

**DOI:** 10.1186/s13068-017-0931-9

**Published:** 2017-10-17

**Authors:** Yongguang Jiang, Peng Xiao, Qing Shao, Huan Qin, Zhangli Hu, Anping Lei, Jiangxin Wang

**Affiliations:** 1 Shenzhen Key Laboratory of Marine Bioresource and Eco-environmental Science, Shenzhen University, Shenzhen, 518060 People’s Republic of China; 2 College of Life Sciences and Oceanography, Shenzhen University, Shenzhen, 518060 People’s Republic of China; 3 Shenzhen Engineering Laboratory for Marine Algal Biotechnology, Shenzhen, 518060 People’s Republic of China; 4 Nanshan District Key Lab for Biopolymers and Safety Evaluation, Shenzhen University, Shenzhen, 518060 People’s Republic of China

**Keywords:** Ethanol, Butanol, Proteomics, *Chlamydomonas*, Microalgae

## Abstract

**Background:**

Microalgae have been demonstrated to be among the most promising phototrophic species for producing renewable biofuels and chemicals. Ethanol and butanol are clean energy sources with good chemical and physical properties as alternatives to gasoline. However, biosynthesis of these two biofuels has not been achieved due to low tolerance of algal cells to ethanol or butanol.

**Results:**

With an eye to circumventing these problems in the future and engineering the robust alcohol-producing microalgal hosts, we investigated the metabolic responses of the model green alga *Chlamydomonas reinhardtii* to ethanol and butanol. Using a quantitative proteomics approach with iTRAQ-LC–MS/MS technologies, we detected the levels of 3077 proteins; 827 and 730 of which were differentially regulated by ethanol and butanol, respectively, at three time points. In particular, 41 and 59 proteins were consistently regulated during at least two sampling times. Multiple metabolic processes were affected by ethanol or butanol, and various stress-related proteins, transporters, cytoskeletal proteins, and regulators were induced as the major protection mechanisms against toxicity of the organic solvents. The most highly upregulated butanol response protein was Cre.770 peroxidase.

**Conclusions:**

The study is the first comprehensive view of the metabolic mechanisms employed by *C. reinhardtii* to defend against ethanol or butanol toxicity. Moreover, the proteomic analysis provides a resource for investigating potential gene targets for engineering microalgae to achieve efficient biofuel production.

**Electronic supplementary material:**

The online version of this article (doi:10.1186/s13068-017-0931-9) contains supplementary material, which is available to authorized users.

## Background

Renewable biofuels are the obvious alternatives to fossil fuels in order to promote sustainable development of the economy and society as well as to reduce carbon dioxide emission [1]. The first generation of biofuels was produced from terrestrial crops such as corn, sugarcane, and rapeseed [2, 3]. However, excessive consumption of these agricultural products may pose a significant threat to the food supply around the world. Therefore, various forms of lignocellulosic biomass were used in the production of the second generation of biofuels [2]. Nonetheless, there is still possible consequence of forest destruction or competing land use. Microalgae are photosynthetic microorganisms that can grow using carbon dioxide as sole carbon source and with minimal nutrient requirements. Diverse microalgal species have been found to be of high photoefficiency and productivity, greatly exceeding that of agricultural crops. Thus, microalgae are attractive cell factories that can produce large amounts of carbohydrates and lipids during short periods of cultivating time in a relatively small water area without competing for arable land [1, 4, 5]. The algal biomass can be processed in place of terrestrial plants for renewable energy production, namely the third generation biofuels [2].

Based on the advances in genome annotation and genetic engineering of microalgae, the fourth generation of biofuels has been suggested to be produced through metabolic engineering of microalgae [6, 7]. Another economic strategy is to allow fuels or precursors to be directly secreted into the growth medium by manipulating the biology of algal cells as reported for *Saccharomyces cerevisiae* [8]. Secretion is more feasible for low molecular weight and hydrophobic compounds, such as hydrogen, alkanes, ethanol, and butanol. The production of hydrogen and alkanes have been reported in microalgae [9, 10], but the biosynthesis of ethanol or butanol has not been explored in this kind of organism.

As alternatives to gasoline, ethanol and butanol have been on the list of desirable biofuels of all generations [11–13]. Ethanol is currently the most common renewable biofuel produced from fermentation of food starches or lignocellulose by *S. cerevisiae* or *Zymomonas mobilis*. Considering the drawbacks of traditional biofuel production processes, it was attractive to directly couple ethanol production to carbon fixation in photosynthetic microorganisms, including cyanobacteria and eukaryotic microalgae [4]. In a previous study, ethanol synthesis was obtained by introducing pyruvate decarboxylase and alcohol dehydrogenase genes from *Z. mobilis* into a unicellular cyanobacterium, *Synechococcus* [14], indicating the feasibility of constructing an integrated system for converting solar energy and inorganic carbon source into biofuel directly. Butanol is also an industrialized biofuel with high energy density and good storage property compared with ethanol. Besides, butanol has been produced from the fermentation of sugar or starch using solventogenic clostridial strains [15]. Because bacteria of the genus *Clostridium* usually has a low growth rate and is difficult to be genetically manipulated, the butanol biosynthesis pathway has been introduced into *S. cerevisiae* [16], *Escherichia coli* [17] and *Synechococcus* [18] for further research. It is also promising to create similar metabolic pathways for ethanol or butanol production in microalgae. The obvious problem for alcohol bioproduction is that, as organic solvents, ethanol and butanol are toxic to microbes including the producers [19]. Therefore, it is important to select highly tolerant microbial strains for industrial production of these biofuels.

Both omics methods and genetic manipulation have been used to examine the organic solvent tolerance of microbes [20–25]. By quantitative transcriptomic analysis and mutant examination, two genes (i.e., slr0724 and sll1392) were validated to be involved in ethanol resistance of *Synechocystis* [26]. Based on the results of transcriptomic and proteomic analysis, a novel regulator Slr1037 and three butanol response genes: sll0690, slr0947, and slr1295 have been explored by gene knockout experiments and were found to be involved in butanol resistance in *Synechocystis* [27, 28]. Ethanol tolerance of *S. cerevisiae* was enhanced by mutagenesis of the TATA-binding protein gene [29]. Improvement in isobutanol tolerance was observed in *E. coli* by simultaneous disruption of five unrelated genes [30]. Together these studies hint at the complexity of microbial resistance mechanisms in response to the stress of a single biofuel. Therefore, the tolerance mechanisms of microalgae should be fully investigated before optimal engineering this organism for ethanol or butanol production can take place.

*Chlamydomonas reinhardtii* is currently used as a model alga for revealing molecular mechanisms of biofuel production in eukaryotic microalgae [31] and various molecular techniques have been developed to manipulate the nuclear and organellar genomes of this organism [32]. In this study, a quantitative proteomics method with isobaric tag for relative and absolute quantitation (iTRAQ) technique and liquid chromatography-tandem mass spectrometry (LC–MS/MS) was applied to explore the global metabolic responses of *C. reinhardtii* under ethanol or butanol exposure. Protein annotation, gene ontology (GO), and KEGG-enrichment analysis were performed to find response proteins. The expression differences of proteins were further verified by real-time quantitative reverse transcription PCR (qRT-PCR).

## Results and discussion

### Effects of ethanol and butanol on growth of *C. reinhardtii* CC-849

**Fig. 1:**
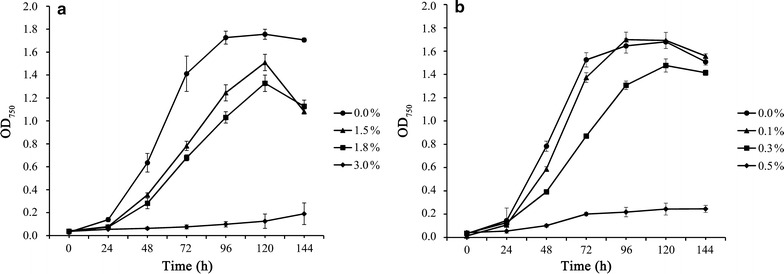
Effects of ethanol (**a**) and butanol (**b**) on the growth of *C. reinhardtii* CC-849

The growth of *C. reinhardtii* CC-849 supplemented with different concentrations of ethanol or butanol was assessed to determine appropriate ethanol or butanol concentrations for proteomic analysis (Fig. 1a, b). All the tested concentrations of ethanol or butanol were found to be inhibitory. The results also showed that 1.8% ethanol and 0.3% butanol, respectively, caused 50% growth decrease at 48 h (corresponding to middle-exponential phase), and therefore these two concentrations were selected for further analysis in this study. The tolerance level of *C. reinhardtii* CC-849 to ethanol was slightly higher than the 1.5% ethanol tolerance level of the cyanobacterium *Synechocystis* sp. PCC 6803 [22] and was much lower than the famously high 25% ethanol tolerance level of *S. cerevisiae* [33]. The tolerance level of *C. reinhardtii* CC-849 to butanol was 50% higher than the 0.2% butanol tolerance level of *Synechocystis* sp. PCC 6803 [23]. However, the butanol tolerance of *C. reinhardtii* CC-849 was weaker than that of the traditional butanol producer *Clostridium acetobutylicum* [21] and other microbial hosts including *E. coli*, *Z. mobilis*, *Pseudomonas putida*, and *S. cerevisiae* [34–36].

Cell morphology in ethanol, butanol, and control treatments were checked under the light microscope, and the results showed that the density of individual cells under ethanol or butanol stress was found to be lower than those of control treatments (Additional file 1: Figure S1). On the contrary, visible aggregation of a large number of cells was found for *Synechocystis* sp. PCC 6803 under ethanol or butanol treated conditions [22, 23]. This morphology likely coincides with the lower tolerance of *Synechocystis* to biofuels than *C. reinhardtii.*

For proteomic analysis, cultivations at 1.8% ethanol, 0.3% butanol, and control treatments were conducted in triplicate. Algal cells were collected at early exponential (24 h), middle exponential (48 h) and exponential-stationary transition (72 h) phases of the cell growth, respectively (Fig. 1a, b). Parallel cell samples were mixed resulting in proteomic samples at three time points for each treatment.

### Overview of quantitative proteomics analysis

**Fig. 2:**
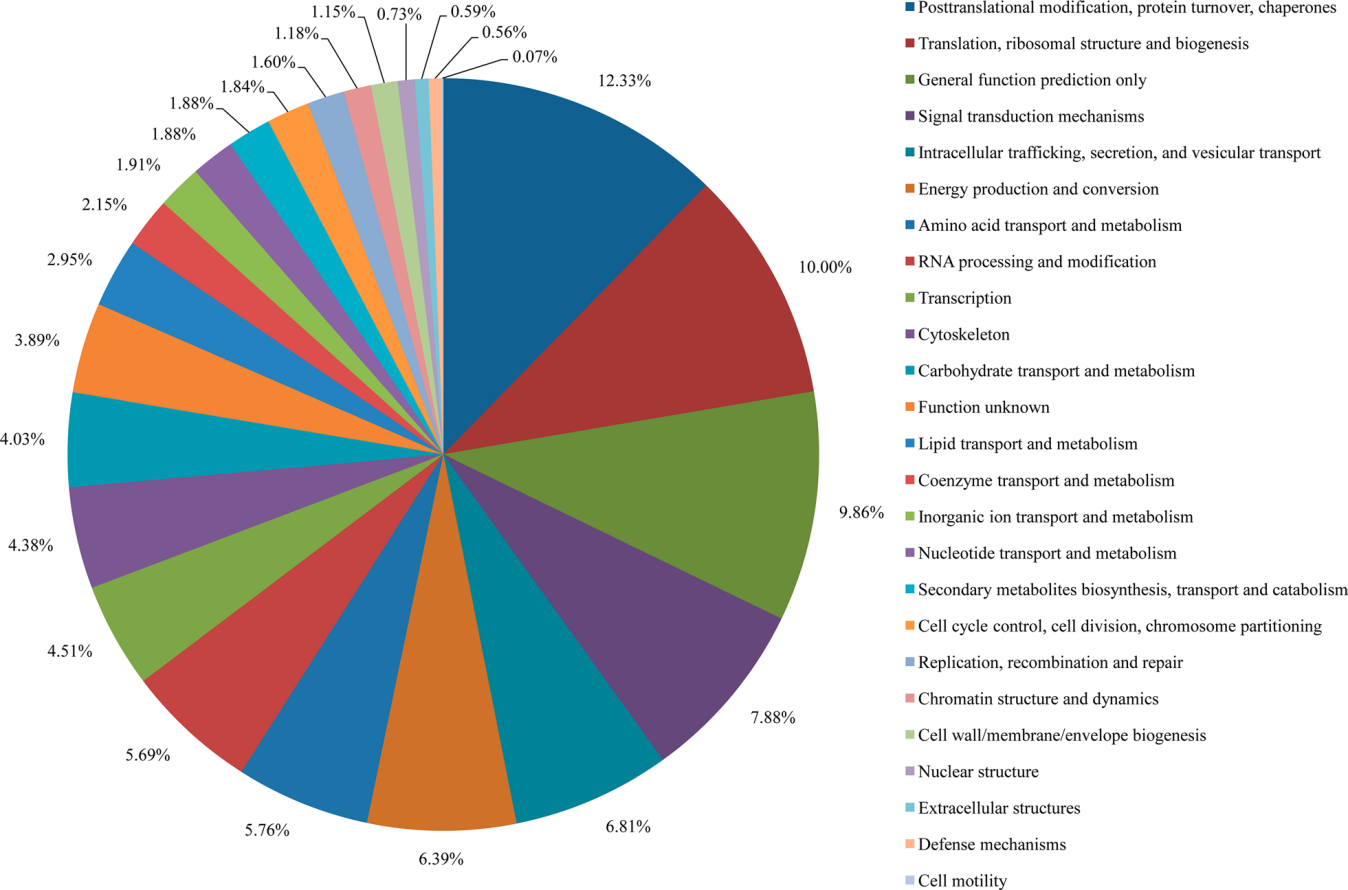
KOG coverage of the proteins detected

After data filtering to eliminate peptides without labeling and reverse-matching peptides, a total of 3077 unique proteins were detected (Additional file 2: Table S1), representing approximately 20% of the 15,143 predicted proteins in the draft genome of *C. reinhardtii* CC-503 [37]. This percentage was close to the overlapping proteome coverage of 25% using several genome annotation databases in a previous study [38], suggesting the methodology we used in the study is reliable. Analysis on eukaryotic cluster of orthologous groups of proteins (KOG) classified 2301 proteins into 25 functional categories, covering almost every aspect of *C. reinhardtii* metabolism (Fig. 2). According to the number of unique proteins identified in each functional category, the three most frequently detected functional categories were “posttranslational modification, protein turnover, chaperones”; “translation, ribosomal structure, and biogenesis”; and “general function prediction only,” each representing 12.33, 10.00, and 9.86% of all the proteins identified. Other well-detected functional categories included “signal transduction mechanisms”; “intracellular trafficking, secretion, and vesicular transport”; “energy production and conversion”; “amino acid transport and metabolism”; and “RNA processing and modification,” each representing over 5% of all the proteins identified.

**Fig. 3:**
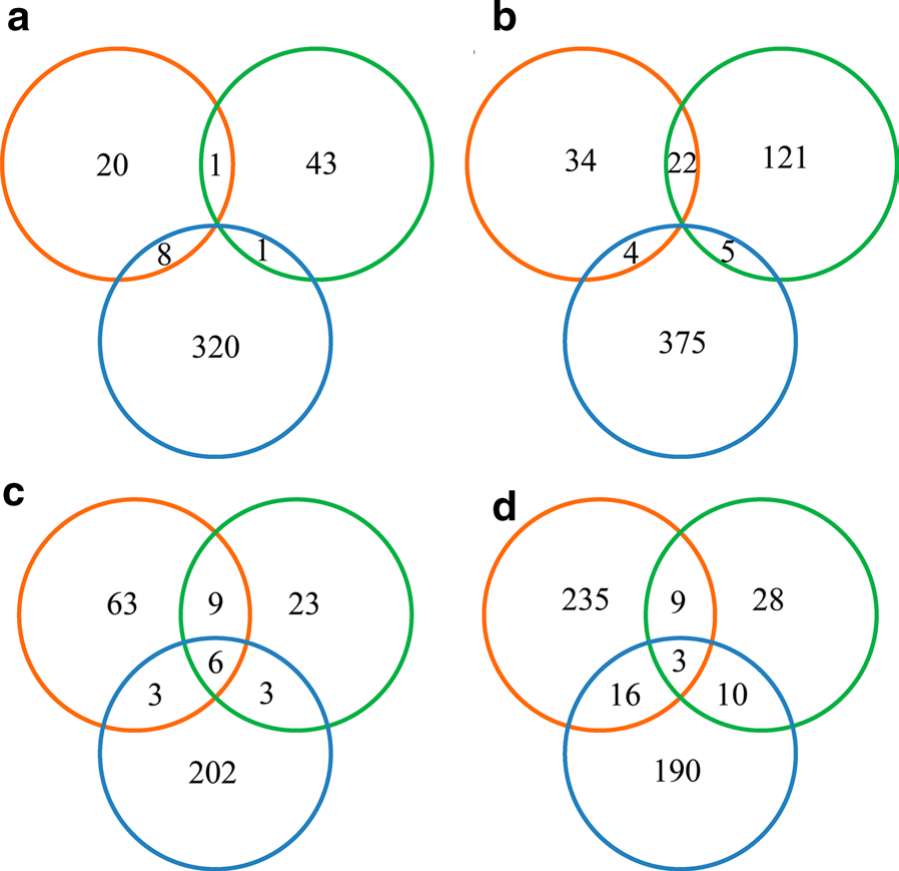
Distribution of upregulated (**a**, **c**) and downregulated (**b**, **d**) proteins at three time points in ethanol (**a**, **b**) and butanol (**c**, **d**) treatments. The three sampling times, 24, 48, and 72 h, are represented by orange, green, and blue circles, respectively

Using a cutoff of 2.0-fold change and a *P* value less than 0.05, we determined that 827 unique proteins were differentially regulated between ethanol and control treatments. The numbers of differential proteins were 89, 193, and 713 at 24, 48, and 72 h, respectively, and far greater differences were observed at 72 h (Fig. 3a, b, Additional file 3: Table S2). During at least two sampling times, 10 and 31 proteins were consistently upregulated and downregulated, respectively (Fig. 3a, b, Table 1).

**Table 1:** Differentially regulated proteins in ethanol treatment

UniProtKB identifier^a^	Ethanol vs control			Description
	24 h	48 h	72 h	
Upregulated proteins				
A8JID6	2.68	0.41	9.20	Plastidic ADP/ATP translocase, AAA1
A8JG58	2.38		2.33	Hypothetical protein
A8JC09	2.36		2.15	Flagellar-associated protein, FAP151
A8IKQ0	2.36		9.46	Fructose-1, 6-bisphosphatase, FBP1
A8IL29	2.21		3.53	Small ARF-related GTPase, ARFA1A
P22675	2.19		4.13	Argininosuccinate lyase, ARG7
Q5NKW4	2.09	2.47	0.25	Photosystem I reaction center subunit II, PsaD
Q7X7A7	2.07		9.04	14-3-3-like protein, Erb14
P06007	2.01		8.87	Photosystem II protein D2, PsbD
A8IRL8		6.61	3.16	Hypothetical protein
Downregulated proteins				
A8JDR3	0.46	0.41	3.13	Ribonucleoside-diphosphate reductase, RIR1
A8J567	0.45	0.48	4.13	Cytosolic ribosomal protein L7a, RPL7a
A8IZV9	0.45		0.11	Flagellar-associated protein, FAP102
A8JDV9	0.45	0.48	11.59	F1F0 ATP synthase gamma subunit, ATP3
A8IVE2	0.45	0.46		Cytosolic ribosomal protein L7, RPL7
A8IVP7	0.44		0.42	Predicted protein
A8J9E9	0.43		0.48	Carotenoid isomerase
Q1WLZ0	0.43	0.48	14.59	60S ribosomal protein L11
A8JAG1	0.41		0.28	Hypothetical protein
A8J387	0.41	0.43	16.14	Hypothetical protein
A8HVQ1	0.39	0.47	16.00	Cytosolic ribosomal protein S8, RPS8
A8I7P5	0.38	0.41	6.49	Magnesium chelatase subunit H, CHLH1
A8HVM3	0.36	0.46	8.87	Hypothetical protein
A8HS48	0.34	0.15	20.32	40S ribosomal protein S3a, RPS3a
E3SC57	0.34	0.19	18.88	60S ribosomal protein L3, RPL3
A8JHB4	0.32	0.33	7.94	Ferredoxin-dependent glutamate synthase, GSF1
A8J9F3	0.32	0.31	16.00	Hypothetical protein
A8IWJ5	0.29	0.46	11.70	Predicted protein
A8IGY1	0.29	0.22	13.68	Cytosolic ribosomal protein S13, RPS13
A8J914	0.27	0.47		UDP-glucose dehydrogenase, UGD2
A8HMG7	0.26	0.34	12.47	Cytosolic ribosomal protein L26, RPL26
A8JGX5	0.25	0.36	16.00	Protein arginine *N*-methyltransferase, PRMT2
A8I647	0.24	0.47	7.94	Zeta-carotene desaturase, ZDS1
A8HYU2	0.23	0.49		Vacuolar H^+^ ATPase V1 sector, subunit C, ATPvC
A8JHC3	0.21	0.29	9.38	Cytosolic ribosomal protein S11, RPS11
A8J8M5	0.21	0.25	10.47	Plastid ribosomal protein S5, PRPS5
A8HQP0		0.46	0.38	Transaldolase, TAL1
A8ILP2		0.48	0.19	Predicted protein
A8J9H8		0.13	0.15	Nucleoside diphosphate kinase, FAP103
Q2HZ22		0.32	0.07	Putative ferredoxin, FDX5
Q42690		0.45	0.20	Chloroplastic fructose-bisphosphate aldolase 1, FBA3

^a^Proteins upregulated or downregulated during at least two sampling times are displayed

Comparison between butanol and control treatments revealed that 730 unique proteins were differentially regulated. The numbers of differential proteins were 344, 91, and 433 at 24, 48, and 72 h, respectively (Fig. 3c, d, Additional file 4: Table S3). During at least two sampling times, 21 and 38 proteins were consistently upregulated and downregulated, respectively (Fig. 3c, d, Table 2). Only one downregulated hypothetical protein (A8IVP7) was shared between ethanol and butanol treatments (Tables 1 and 2), indicating different metabolic responses to these two organic solvents in *C. reinhardtii*.

**Table 2:** Differentially regulated proteins in butanol treatment

UniProtKB identifier^a^	Butanol vs control			Description
	24 h	48 h	72 h	
Upregulated proteins				
A8J7T7	6.03		2.44	Cysteine endopeptidase, CEP1
L8B958	4.49	3.11		Pyruvate ferredoxin oxidoreductase, PFO
Q66YD0	4.41	3.08		Chloroplast vesicle-inducing protein in plastids 1, VIPP1
O64925	4.06	3.91	2.63	Granule-bound starch synthase, STA2
A8JHZ9	3.66	5.30	2.27	Hypothetical protein
A8I980	3.47	2.51	2.36	Delta-aminolevulinic acid dehydratase, ALAD
O49822	3.44	2.61		Ascorbate peroxidase, Apx1
A8IX35	2.91	2.36	6.61	Hypothetical protein
A8J0W9	2.88	2.29		NAD-dependent malate dehydrogenase, MDH3
A8JHP0	2.78	2.17		Oxidoreductase-like protein, CPLD35
A8J6Y3	2.70	2.91	2.03	Hypothetical protein
A8J000	2.58	2.23		Acetate kinase, ACK1
A8IYS5	2.54	2.94	2.42	Septin-like protein, SEP1
A8I0K9	2.33	3.28		Dehydroascorbate reductase, DHAR
A8IZU0	2.29	2.05		Heat shock protein 70C, Hsp70C
A8HVU5	2.27		2.36	Phosphoglycerate mutase, PGM1a|PGM1b
Q9ZSJ4	2.19		4.02	Light-harvesting complex II protein, Lhcb3
O22448	2.01	3.66		Glutathione peroxidase homolog, Gpxh
A8IUI1		3.91	2.75	Hypothetical protein
A8JBW0		3.44	2.91	Hypothetical protein
A8JGL7		3.98	38.37	Heme peroxidase-related protein, Cre.770
Downregulated proteins				
A8J597	0.49		0.29	Cytosolic ribosomal protein L12, RPL12
A8IZ36	0.47		0.16	Cytosolic ribosomal protein S25, RPS25
A8JGI9	0.46		0.33	Cytosolic ribosomal protein S7, RPS7
A8JE91	0.46	0.40		Chaperonin 60B1, CPN60B1
A8HTY0	0.44		0.09	Plastid ribosomal protein L7/L12, PRPL7/L12
A8JIB7	0.43	0.49		Chaperonin 60A, CPN60A
A8IAN1	0.40	0.47	0.15	Transketolase, TRK1
A8J768	0.39		0.28	Ribosomal protein S14, RPS14
A8JDM1	0.39	0.34		Hypothetical protein
A8IV98	0.39		0.36	DEAD box RNA helicase
A8J503	0.38		0.24	Plastid ribosomal protein L6, PRPL6
A8IVZ9	0.36		0.32	Glutamine synthetase, GLN2
A8JAP7	0.34		0.20	Hypothetical protein
A8IAT4	0.34	0.47	0.24	Acetohydroxy acid isomeroreductase, AAI1
A8JCQ8	0.33	0.34		Acetyl CoA synthetase, ACS2
A8J9D9	0.33		0.38	Plastid ribosomal protein L24, PRPL24
A8I403	0.33		0.19	Cytosolic ribosomal protein S19, RPS19
A8JFR9	0.30	0.23	0.28	Acetyl CoA synthetase, ACS3
A8J785	0.30		0.14	Chloroplastic ATP synthase subunit b’, ATPG
A8J841	0.29	0.48		Hydroxymethylpyrimidine phosphate synthase, THICb|THICa
A8IVS6	0.28	0.40		Hypothetical protein
A8ITX0	0.28	0.46		Copper response target 1 protein, CRD1
A8IUV7	0.27		0.36	Cytosolic ribosomal protein L13, RPL13
A8JGT1	0.24		0.15	RNA helicase, RHE
A8IW20	0.22	0.49		Eukaryotic initiation factor, EIF4G
A8I0R6	0.22		0.47	Hypothetical protein
A8HP90	0.19		0.39	Cytosolic ribosomal protein L6, RPL6
A8I9M5	0.17	0.31	10.86	Hypothetical protein
A8HUK0		0.45	0.28	Peptidyl-prolyl *cis*–*trans* isomerase, FKB12
A8I495		0.39	0.38	Obg-like ATPase 1, OLA1
A8IK91		0.45	0.34	Translocon component Tic40-related protein, TIC40
A8IPS8		0.35	0.16	Hypothetical protein
A8IVP7		0.26	0.43	Hypothetical protein
A8J2S0		0.29	0.43	Citrate synthase, CIS2
A8J6J6		0.34	0.18	Acetyl-CoA acyltransferase, ATO1
A8JCW5		0.40	0.38	Hypothetical protein
A8JHB7		0.33	0.18	Hypothetical protein
Q9ZTA7		0.49	0.33	Protoporphyrinogen oxidase, Ppx1

^a^Proteins upregulated or downregulated during at least two sampling times are displayed

### qRT-PCR validation of the proteomic analysis

**Fig. 4:**
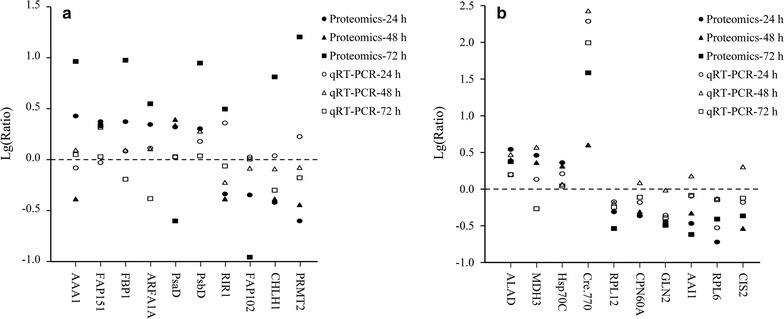
Comparison of ratios calculated from proteomic and qRT-PCR analyses. **a** Ethanol treatment; **b** butanol treatment. The values were calculated by lg(Ratio)

To examine the reliability of iTRAQ analysis, a subset of 20 genes were selected for qRT-PCR analysis, ten genes for ethanol and butanol treatments, respectively. These genes were chosen based on the expression levels of their corresponding proteins. Among them, ten proteins were upregulated (i.e., AAA1, FAP151, FBP1, ARFA1A, PsaD, PsbD, ALAD, MDH3, Hsp70C, and Cre.770), and ten proteins were downregulated (i.e., RIR1, FAP102, CHLH1, PRMT2, RPL12, CPN60A, GLN2, AAI1, RPL6, and CIS2) according to the proteomic analyses. As displayed in Fig. 4, the values higher than zero represents upregulation and those lower than zero represents downregulation. The RNA level of differential proteins varied with the same pattern to the regulation of protein level during at least two sampling times. Although the correlation between RNA expression and protein abundance is usually very weak, visible positive correlation was observed between proteomic analysis and qRT-PCR analysis, suggesting an overall good quality of the iTRAQ results.

### Enrichment of GO terms

GO analysis was performed by matching the identified proteins to the proteins annotated with GO terms, and 2310 proteins were annotated (Additional file 5: Table S4). GO enrichment analysis was then carried out to determine the affected cellular metabolisms. As a result, 129 and 126 unique GO terms were enriched for the ethanol and butanol treatments, respectively (Additional file 6: Table S5 and Additional file 7: Table S6). For ethanol treatment, 21 GO terms were shared by at least two time points, including six terms for molecular function and 15 terms for biological process (Table 3). The results showed that three main categories of molecular functions, including “structural constituent of ribosome”; “structural molecule activity”; and “ligase activity, forming carbon–oxygen bonds, aminoacyl-tRNA, and related compounds” were affected by ethanol. Several key biological processes, including “translation and its regulation,” “amino acid activation and tRNA aminoacylation,” “amino acid metabolic process,” “protein metabolic process and its regulation,” and “macromolecule and organic substance biosynthetic processes” were also confirmed to be affected by ethanol. Therefore, protein biosynthesis was most seriously affected by ethanol. In a previous RNA-seq study, genes related with protein biosynthesis were also found to be affected by ethanol in *Synechocystis* sp. PCC6803 [26].

**Table 3:** GO enrichment analysis of differentially regulated proteins in ethanol treatment

GO term^a^	GO ID	*P* value^b^		
		24 h	48 h	72 h
Molecular function				
Structural constituent of ribosome	GO:0003735	0.002	0.003	0.000
Aminoacyl-tRNA ligase activity	GO:0004812	0.000	0.011	
Structural molecule activity	GO:0005198	0.001	0.000	0.000
Ligase activity	GO:0016874	0.002	0.002	
Ligase activity, forming carbon–oxygen bonds	GO:0016875	0.000	0.011	
Ligase activity, forming aminoacyl-tRNA and related compounds	GO:0016876	0.000	0.011	
Biological process				
Translation	GO:0006412	0.000	0.000	0.000
Regulation of translation	GO:0006417		0.002	0.015
Cellular amino acid metabolic process	GO:0006520	0.008		0.032
Metabolic process	GO:0008152	0.042		0.005
Biosynthetic process	GO:0009058	0.046		0.001
Macromolecule biosynthetic process	GO:0009059	0.007	0.002	0.003
Protein metabolic process	GO:0019538	0.001	0.002	0.000
Regulation of cellular protein metabolic process	GO:0032268		0.008	0.005
Cellular macromolecule biosynthetic process	GO:0034645	0.007	0.002	0.003
Amino acid activation	GO:0043038	0.000	0.010	
tRNA aminoacylation	GO:0043039	0.000	0.010	
Cellular metabolic process	GO:0044237	0.043		0.000
Cellular protein metabolic process	GO:0044267	0.001	0.001	0.000
Regulation of protein metabolic process	GO:0051246		0.018	0.008
Organic substance biosynthetic process	GO:1901576	0.032		0.000

^a^GO terms present during at least two sampling times are displayed

^b^ *P* values were calculated by Chi-square test

For butanol treatment, 22 GO terms were shared by at least two time points, including seven terms for molecular function and 15 terms for biological process (Table 4). The results revealed that six main categories of molecular functions, including “ribonucleoside binding,” “adenyl ribonucleotide binding,” “structural constituent of ribosome,” “structural molecule activity,” “rRNA binding,” and “tetrapyrrole binding” were affected by butanol. Several key biological processes, including “gene expression”; “translation”; “protein, monosaccharide, hexose, and organic substance metabolic process”; and “macromolecule and organic substance biosynthetic process” were also confirmed to be affected by butanol. Effects of butanol on the “structural molecule activity,” “RNA binding,” and “gene expression processes” were also reported for *Synechocystis* sp. PCC6803 [23].

**Table 4:** GO enrichment analysis of differentially regulated proteins in butanol treatment

GO term^a^	GO ID	*P* value^b^		
		24 h	48 h	72 h
Molecular function				
Nucleoside binding	GO:0001882	0.043		0.020
Structural constituent of ribosome	GO:0003735	0.000		0.000
Structural molecule activity	GO:0005198	0.000		0.000
rRNA binding	GO:0019843	0.035		0.028
Ribonucleoside binding	GO:0032549	0.043		0.020
Adenyl ribonucleotide binding	GO:0032559	0.045		0.003
Tetrapyrrole binding	GO:0046906	0.042		0.007
Biological process				
Monosaccharide metabolic process	GO:0005996		0.033	0.019
Translation	GO:0006412	0.000		0.000
Metabolic process	GO:0008152	0.042		0.024
Biosynthetic process	GO:0009058	0.000		0.004
Macromolecule biosynthetic process	GO:0009059	0.000		0.002
Gene expression	GO:0010467	0.000		0.049
Hexose metabolic process	GO:0019318		0.029	0.015
Protein metabolic process	GO:0019538	0.000		0.000
Cellular macromolecule biosynthetic process	GO:0034645	0.000		0.002
Cellular metabolic process	GO:0044237	0.002		0.009
Primary metabolic process	GO:0044238	0.009		0.006
Cellular biosynthetic process	GO:0044249	0.000		0.004
Cellular protein metabolic process	GO:0044267	0.000		0.000
Organic substance metabolic process	GO:0071704	0.003		0.021
Organic substance biosynthetic process	GO:1901576	0.000		0.001

^a^GO terms present during at least two sampling times are displayed

^b^ *P* values were calculated by Chi-square test

### Enrichment of KEGG pathways

KEGG analysis was performed to reveal the affected metabolic pathways. For ethanol treatment, the “ribosome” pathway was significantly affected in three growth phases, which coincided with the enriched GO terms and suggesting that this pathway was very active upon ethanol exposure (Table 5). In addition, the “aminoacyl-tRNA biosynthesis” pathway was active in early-exponential phase, while the “oxidative phosphorylation” and “porphyrin and chlorophyll metabolism” pathways were active in the exponential-stationary transition phase. For butanol treatment, the “ribosome,” “microbial metabolism in diverse environments,” “carbon metabolism,” and “carbon fixation in photosynthetic organisms” pathways were significantly affected in at least two growth phases, suggesting that these pathways were very active under butanol stress (Table 5). In addition, the “biosynthesis of secondary metabolites,” “porphyrin and chlorophyll metabolism,” and “RNA degradation” pathways were active in middle-exponential phase, while the “biosynthesis of amino acids,” “photosynthesis,” and “methane metabolism” pathways were active in exponential-stationary transition phase. Association of the ethanol- or butanol-responsive proteins with multiple GO terms and KEGG pathways suggested that algal cells employ multiple and synergistic mechanisms in resistance to single biofuel stress. Adaptations in a diversity of cellular processes were also observed in evolved isobutanol-tolerant *E. coli* strains [39] and a mutant *C. acetobutylicum* strain with enhanced butanol tolerance and yield [21].

**Table 5:** KEGG pathway enrichment analysis of differentially regulated proteins in ethanol and butanol treatments

Pathway term	Pathway ID	Protein number			*P* value^a^		
		24 h	48 h	72 h	24 h	48 h	72 h
Ethanol treatment							
Ribosome	ko03010	11	17	58	0.007	0.012	0.000
Aminoacyl-tRNA biosynthesis	ko00970	8			0.000		
Oxidative phosphorylation	ko00190			32			0.011
Porphyrin and chlorophyll metabolism	ko00860			20			0.011
Butanol treatment							
Ribosome	ko03010	53		42	0.000		0.000
Biosynthesis of secondary metabolites	ko01110		16			0.015	
Microbial metabolism in diverse environments	ko01120		10	43		0.026	0.034
Carbon metabolism	ko01200		9	38		0.014	0.010
Porphyrin and chlorophyll metabolism	ko00860		4			0.019	
Carbon fixation in photosynthetic organisms	ko00710		4	13		0.027	0.046
RNA degradation	ko03018		4			0.011	
Biosynthesis of amino acids	ko01230			33			0.039
Photosynthesis	ko00195			18			0.005
Methane metabolism	ko00680			11			0.041

^a^ *P* values were calculated by Chi-square test

### Butanol affects stress-related proteins

Exposure to organic solvent is an environmental stress to microbes such as *E. coli*, *C. acetobutylicum*, *Synechocystis*, *Z. mobilis* and *S. cerevisiae* [19, 22, 23, 40–42]. Prokaryotes and eukaryotes may respond to environmental stress by upregulation of heat shock proteins (Hsps). Previous studies have revealed that Hsps were relevant to ethanol or butanol tolerance in *Synechocystis* [22, 23]. The Hsps are chaperons that mediate the correct folding of proteins to prevent the aggregation of misfolded proteins and repair intracellular injury during a variety of stress conditions [43]. In this study, Hsp70C was upregulated in *C. reinhardtii* under butanol exposure. Hsp70C is localized to mitochondria and is inducible by heat shock and light [44]. In contrast, Chaperonin 60A and Chaperonin 60B1 were downregulated by butanol treatment. Cpn60A and Cpn60B1 could be induced by heat shock conditions in *C. reinhardtii* [45] and they are required for the formation of a normal plastid division apparatus in *Arabidopsis thaliana* [46].

It was reported that ethanol and butanol are toxic to microbial cells (i.e. *Synechocystis*, *E. coli*, and *S. cerevisiae*) as they induce production of reactive oxygen species and oxidative stress [22, 23, 47, 48]. Our proteomic analysis showed that oxidative stress response was also induced in *C. reinhardtii* by butanol. The upregulated antioxidant enzymes were ascorbate peroxidase Apx1, dehydroascorbate reductase DHAR, glutathione peroxidase homolog Gpxh and heme peroxidase-related protein Cre.770. Apx1 is a hydrogen peroxide-scavenging enzyme that is specific to plants and algae, and is found to be indispensable to protect chloroplasts and other cell constituents from oxidative damage [49]. For instance, Apx1 plays an important role in protecting *C. reinhardtii* against oxidative damage imposed by salt stress [50]. In addition, DHAR catalyzes the reduction of dehydroascorbate to ascorbate and would provide sufficient substrate for Apx1. It has been reported that the tolerance of tobacco to ozone and drought stresses was largely enhanced by overexpression of DHAR in its cytosol [51]. Gpxh is a potential isoenzyme of glutathione peroxidase that plays a key role in antioxidant system of various microbes [22, 23, 48, 52]. Similarly, Cre.770 is probable a heme-containing peroxidase, which was greatly induced at both the protein and RNA levels (Table 2 and Fig. 4b), indicating an important role in resistance to oxidative stress in *C. reinhardtii*.

### Ethanol and butanol affect transporters and cytoskeleton-related proteins

Membrane transporters have been suggested to be one important mechanism against toxic chemicals by microbes [19, 43, 53]. Several transporters are involved in tolerance to ethanol, butanol, and hexane in *Synechocystis* [22, 23, 25, 40]. In the presence of ethanol, we found the plastidic ADP/ATP translocase AAA1 and a potential transporter FAP151 were upregulated. AAA1 exchanges plastid ADP for ATP present in the eukaryotic cytoplasm [54] and the induction of this protein implies a compensate mechanism for ATP supply in chloroplasts when photophosphorylation was inhibited by alcohol. FAP151 contains two conserved domains of ABC transporters that are ubiquitous membrane proteins coupling the transport of diverse substrates across cellular membranes to the hydrolysis of ATP [55]. In addition, the vacuolar H^+^ ATPase ATPvC was downregulated by ethanol treatment. ATPvC is a universal proton pump of eukaryotes and is required for endocytic and secretory trafficking in *Arabidopsis* [56, 57]. A flagellar-associated protein FAP102 was downregulated by ethanol treatment but the function of FAP102 is still unknown. A septin-like protein SEP1 was upregulated by butanol treatment, and this protein functions in cells by localizing other proteins to special cell sites, indicating improvement of intracellular trafficking under butanol stress. Another investigation also showed a clear induction of proteins involved in intracellular movements in *C. reinhardtii* growing on metal-rich natural acidic water [58].

### Ethanol and butanol affect the regulatory systems

Responses of microbes to environmental stress were often mediated by regulatory systems such as two-component systems, kinases, GTPases, methylases, and transcriptional regulators [22, 23, 25, 59]. After ethanol treatment of *C. reinhardtii*, the small ADP-ribosylation factor-related GTPase ARFA1A and 14-3-3-like protein Erb14 were upregulated, while the protein arginine *N*-methyltransferase PRMT2 was downregulated. The homologous protein of ARFA1A in mouse regulates protein trafficking between intracellular organelles and is essential for lipid droplet growth in adipose cells [60]. Members of the Erb14 family have been identified as regulatory elements in intracellular signaling pathways and cell cycle control [61]. Protein methylation at arginine or lysine is an important posttranslational modification and methylation of eyespot proteins of *C. reinhardtii* influences the size and position of the eyespot [62]. PRMTs are a family of enzymes that function by transferring a methyl group to the guanidine group of arginine residues in target proteins, and the most notable is the PRMT-mediated methylation of histone proteins, which causes chromatin remodeling and influencing gene transcription. The physiological role of PRMT2 is still unknown while PRMT1 was reported to modulate light-harvesting antenna translation in *C. reinhardtii* [63]. Butanol treatment of *C. reinhardtii* caused downregulation of peptidyl-prolyl *cis*–*trans* isomerase FKB12, translocon component Tic40-related protein TIC40 and Obg-like ATPase 1 OLA1. *Chlamydomonas* FKB12 exhibits high affinity to rapamycin in vivo and is a signal protein mediating rapamycin action to inhibit cell growth [64, 65]. Thus, downregulation of FKB12 may induce the cell resistance to a toxic metabolite. TIC40 may function as a co-chaperone that facilitates protein translocation across the inner membrane [66]. OLA1 is a cytosolic ATPase, and multiple roles of this protein have been reported, for instance, protecting mammalian cells by stabilizing Hsp70 during heat shock [67].

### Ethanol and butanol affect photosynthesis

The photosystem of photosynthetic microbes are vulnerable under stress conditions such as salt, heavy metals, and organic solvents [58, 68, 69]. However, in previous investigations, a set of photosynthesis-related proteins were upregulated by ethanol or butanol treatment in *Synechocystis* [22, 23, 25]. Interestingly, we also found the photosystem I reaction center subunit PsaD and the photosystem II protein PsbD were upregulated by ethanol treatment in *C. reinhardtii*. In contrast, four photosynthesis-related proteins were downregulated by ethanol treatment, including magnesium chelatase subunit CHLH1 involved in chlorophyll biosynthesis [70], zeta-carotene desaturase ZDS1 involved in carotenoid biosynthesis, carotenoid isomerase, and putative ferredoxin FDX5. Carotenoid isomerase was previously found to be involved in photoprotection in rice [71] and may has a similar function in *C. reinhardtii*. FDX5 plays a critical role in maintaining thylakoid membrane structure and dark metabolism of *C. reinhardtii* [72].

In the case of butanol treatment, three photosynthesis-related proteins were upregulated, including chloroplast vesicle-inducing protein VIPP1, light-harvesting complex II protein Lhcb3, and delta-aminolevulinic acid dehydratase ALAD. VIPP1 is essential for the biogenesis of thylakoid membranes [73] in *Chlamydomonas* and photosystem I in *Synechococcus* [74]. Its induction is beneficial for maintaining the integrity of photosynthetic system. ALAD is a chlorophyll biosynthetic enzyme, and its activity could be induced by light [75]. Two photosynthetic proteins were downregulated by butanol treatment, including copper response target 1 protein CRD1 involved in chlorophyll biosynthesis [76] and protoporphyrinogen oxidase Ppx1, which is involved in porphyrin and chlorophyll metabolism [77].

### Ethanol and butanol affect protein synthesis

A set of ribosomal proteins were significantly downregulated by ethanol or butanol treatment, which explains the slow growth of *C. reinhardtii* under these stress conditions. Reduction of ribosomal proteins was also observed for *Synechocystis* when exposed to ethanol, butanol, and hexane [22, 23, 40], suggesting common resistance mechanism used by different microbes against environmental stress, such as adopting slowdown of protein biosynthesis and slower growth. Similarly, the protein synthesis rate was found to be decreased in *C. reinhardii* under salt stress [78]. We found several proteins involved in amino acid biosynthesis were also downregulated, such as ferredoxin-dependent glutamate synthase GSF1 with ethanol, and glutamine synthetase GLN2 and acetohydroxy acid isomeroreductase AAI1 with butanol. The eukaryotic initiation factor for translation EIF4G was also downregulated by butanol treatment. However, the l-arginine synthetase argininosuccinate lyase ARG7 was upregulated by ethanol treatment.

### Ethanol and butanol affect carbohydrate and ATP metabolism

In a previous investigation, proteins involved in carbohydrate metabolism were mostly unchanged in *Synechocystis* under ethanol stress conditions [22]. However, carbohydrate metabolic processes were significantly affected in *C. reinhardtii* under metal-rich stress conditions [58]. In this study, the fructose-1, 6-bisphosphatase FBP1 was upregulated, while chloroplastic fructose-bisphosphate aldolase 1 FBA3, UDP-glucose dehydrogenase UGD2, and transaldolase TAL1 were downregulated by ethanol treatment. FBP1 is critical in gluconeogenesis and Calvin cycle, and TAL1 is important for the balance of metabolites in the pentose-phosphate pathway.

Under butanol treatment, five proteins involved in carbohydrate metabolism were upregulated, including phosphoglycerate mutase PGM1, NAD-dependent malate dehydrogenase MDH3, acetate kinase ACK1, pyruvate ferredoxin oxidoreductase PFO and granule-bound starch synthase STA2. It was reported that PFO of *C. reinhardtii* was located in chloroplasts and was central to anaerobic energy metabolism through pyruvate decarboxylation and formation of acetyl-coenzyme A with concomitant reduction of low-potential ferredoxins or flavodoxins [79]. Since biosynthesis of butanol is also an anaerobic process, the upregulation of PFO may enhance the energy supply for butanol synthesis. On the other side, five proteins were downregulated by butanol treatment, including citrate synthase, acetyl CoA synthetases, ACS2 and ACS3, acetyl-CoA acyltransferase ATO1, and transketolase TRK1. Two proteins involved in ATP synthesis were downregulated, including F1F0 ATP synthase gamma subunit ATP3 in ethanol treatment and chloroplastic ATP synthase subunit b’ in butanol treatment.

### Ethanol and butanol affects other central metabolic processes

Several proteins involved in other primary metabolism of *C. reinhardtii* were also significantly affected by ethanol or butanol stress. Ribonucleoside diphosphate reductase RIR1 and nucleoside diphosphate kinase FAP103 were downregulated by ethanol. RIR1 catalyzes the biosynthesis of deoxyribonucleotides in the DNA replication pathway and FAP103 is crucial for the homeostasis of cellular nucleoside di- and triphosphate composition and may control many cellular functions. Butanol treatment caused cysteine endopeptidase CEP1 involved in proteolysis and an oxidoreductase-like protein CPLD35 to be upregulated, while hydroxymethylpyrimidine phosphate synthase THICb involved in thiamine biosynthesis and two RNA helicases were downregulated.

### Effects of ethanol and butanol on uninvestigated proteins

Two hypothetical proteins (A8JG58 and A8IRL8) were found upregulated, and seven (A8IVP7, A8JAG1, A8J387, A8HVM3, A8J9F3, A8IWJ5, and A8ILP2) were downregulated by ethanol. Five hypothetical proteins (A8JHZ9, A8IX35, A8J6Y3, A8IUI1, and A8JBW0) were upregulated, while nine (A8JDM1, A8JAP7, A8IVS6, A8I0R6, A8I9M5, A8IPS8, A8IVP7, A8JCW5, and A8JHB7) were downregulated by butanol. These proteins were predicted in genome sequencing project of *C. reinhardtii* [37] and their strong regulation upon the exposure of ethanol or butanol may help to elucidate their functions. The high percentage of functionally unknown proteins detected is not unreasonable, considering more than 71% of proteins in the *C. reinhardtii* genome are still annotated as hypothetical proteins.

## Conclusions

Quantitative proteomics analysis revealed that ethanol or butanol exposure caused significant slowdown of primary metabolisms in *C. reinhardtii*, such as photosynthesis, protein synthesis, carbohydrate, and ATP metabolism, and other central metabolic processes. The expression of stress-related proteins, transporters, cytoskeleton-related proteins, and regulators were upregulated in the algal cells as major protection mechanisms against ethanol or butanol toxicity. In addition, antioxidant enzymes were significantly upregulated by butanol treatment compared with ethanol treatment, implying that butanol may cause strong oxidative stress in *C. reinhardtii*. These metabolic responses are much similar to what has been observed in other microbes, such as *E. coli*, *C. acetobutylicum*, *Z. mobilis*, *Synechocystis,* and *S. cerevisiae* [19, 22, 23, 41, 42], indicating the presence of possible common resistance strategies to ethanol or butanol among various species.

In summary, these data provide the first comprehensive view of metabolic responses employed by a model green alga *C. reinhardtii* to defend against ethanol or butanol stress. This proteomic analysis will provide a resource for investigating potential target genes/proteins for manipulating the algal cells to improve their ethanol or butanol tolerance and thus aid in their biofuel production. Further work will be required to determine the exact relationship between these genes and organic solvent tolerance. The probable heme-containing peroxidase Cre.770 and the pyruvate ferredoxin oxidoreductase PFO are the most promising targets for metabolic engineering of *C. reinhardtii* to achieve efficient biofuel production.

## Methods

### Algal culture conditions and biofuel treatment

The cell wall-deficient strain *C*. *reinhardtii* CC-849 was obtained from the *Chlamydomonas* Genetic Center (Duke University). The strain was cultured in liquid TAP medium [80] at 22 °C by providing continuous white light at a density of 30 μmol m^−2^ s^−1^. For growth and biofuel treatment, 5 mL fresh algal cells at exponential phase (1 × 10^6^ cells mL^−1^) were incubated into 50 mL TAP medium in 250-mL conical flasks. Biofuel was added into the medium at the beginning of cultivation with varying final concentrations, 0.0, 1.5, 1.8, and 3.0% (v/v) for ethanol and 0.0, 0.1, 0.3, and 0.5% (v/v) for butanol. The treatment of each concentration was performed in triplicate, and the 0.0% concentration was used as control treatment. One milliliter culture sample was collected every 24 h over a culture period of 6 days, and the optical density at 750 nm (OD_750_) was measured by NanoDrop 2000 spectrophotometer (Thermo Fisher) for growth monitoring. Analytic purity ethanol and *n*-butanol were purchased from Aladdin (China). Algal cells for proteomic and qRT-PCR analysis were collected at 24, 48, and 72 h. The cells from triplicate samples were mixed and centrifuged at 8000×*g* for 5 min at 4 °C. The cell pellets were rinsed in PBS and stored in liquid nitrogen before protein preparation.

### Protein extraction and digestion

For each sample, cell pellets were resuspended in lysis buffer (8 M urea, 4% CHAPS, 40 mM Tris–HCl, 2 mM EDTA) with 1 mM protease inhibitor PMSF. The algal cells were lysed by vigorously vortexing for 5 min. Then, dithiothreitol was added to the lysis solution at a final concentration of 10 mM and mixed thoroughly. The sample was centrifuged at 20,000×*g* for 30 min, and the supernatant was mixed with ice-cold acetone (1:4, v/v) containing 30 mM dithiothreitol. After repeating the lysis step twice, the supernatants were combined, and proteins were harvested by centrifugation at 10,000×*g* for 30 min after precipitation at − 20 °C overnight. The protein pellets were resuspended in 100 mM triethylammonium bicarbonate solution, and total protein concentration was measured by Bradford assay (Beyotime, China). Before digestion, 200 μg of protein from each lysate was reduced, and a cysteine-blocking reaction was performed using reagents provided in the iTRAQ array kit (AB Sciex, USA). The solution was then filtered through an ultracentrifugal filter (Sartorious, 10 kDa) to remove reagent residues. Proteins remaining on the filter were digested with trypsin (1:50 w/w, AB Sciex) in a 50 μL reaction volume overnight at 37 °C. Peptides were harvested by centrifugation at 12,000×*g* for 20 min. The centrifugation step was repeated after 50 μL dissolution buffer (AB Sciex) was added to the filter and the filtrates were combined.

### iTRAQ labling

The iTRAQ labeling of 100 μg peptides from digestion of each sample was performed using iTRAQ Reagent 8-plex and 4-plex Kit (AB Sciex) according to the manufacturer’s protocol. The peptides were labeled with respective isobaric tags by incubation at room temperature for 2 h and then lyophilized via vacuum centrifugation. The labeled control and biofuel treatment samples were reconstituted in buffer A (20 mM ammonium formate, pH = 10.0), pooled, and fractionated using Durashell-C18 column (250 × 4.6 mm, 5 μm particle size, 100 Å pore size, Agela) by HPLC system at a flow rate of 0.8 mL min^−1^. The HPLC gradient consisted of 95% buffer A for 15 min; 5–15% buffer B (20 mM ammonium formate, pH = 10.0, 80% v/v acetonitrile) for 25 min; 15–38% buffer B for 15 min; 38–90% buffer B for 1 min followed by 90% buffer B for 9 min and 95% buffer A for 5 min. The chromatograms were monitored by ultraviolet absorbance at 218 nm. The fractions were collected every 1 min after the 5th min and 48 fractions were obtained for each injection. The fractions were desalted with Sep-Pak Vac C18 cartridges (Waters, USA), vacuum centrifuged to dryness and reconstituted in buffer C (2% acetonitrile, 0.1% formic acid) for LC–MS/MS analysis.

### LC–MS/MS proteomic analysis

The mass spectroscopy analysis was performed using a TripleTOF 5600 mass spectrometer coupled with online Eksigent nano LC system (AB Sciex, USA). For LC conditions, a nanobored C18 column (15 cm × 75 μm, 5 μm particle size) with a picofrit nanospray tip (New Objectives, USA) and loading pump with a constant flow rate of 2 μL min^−1^ were applied. The peptides were separated by a micro flow rate of 0.3 μL min^−1^ and the injection volume was 8 µL. LC gradient was adjusted according to different peptide fractions, and in general, 95–90% buffer C for 0.1 min; 10–25% buffer D (98% acetonitrile, 0.1% formic acid) for 60 min; 25–48% buffer D for 25 min; 48–80% buffer D for 1 min followed by 80% buffer D for 4 min and 95% buffer C for 10 min. The parameters of mass spectrometer were set as previously described [23]. In brief, positive ions were monitored with selected mass ranges of 350–1250 *m*/*z* for TOF MS scan and 100–1500 *m*/*z* for product ion scan. The peptides with + 2 to + 4 charge states were selected for MS/MS. The ten most abundant ions above a five count threshold in an accumulation period of 0.1 s and a dynamic exclusion period of 25 s were selected for MS/MS. The relative abundance of the proteins in the samples was calculated based on the peak areas of the iTRAQ reporter ions.

### Proteomic data analysis

The MS data were processed using ProteinPilot 4.5 (AB Sciex) software. Peptide identification and protein summarization were performed using the Paragon and Pro Group algorithms implemented in ProteinPilot. The parameters were set as iTRAQ labeling at N-terminal and lysine residues, cysteine modification by methyl methanethiosulfonate and trypsin as a protease. Proteins identified with low false discovery rate (≤ 1%) were used for further analysis. For iTRAQ quantification, the peptides were automatically selected by the software to calculate the reporter peak area. The resulting dataset was auto bias-corrected to eliminate any variations caused by unequal mixing while combining different samples. The MS/MS data were searched against genome annotations of *C. reinhardtii* CC-503 deposited in NCBI database [81]. Proteins with 2.0-fold change between biofuel-treated and control samples and *P* values of statistical evaluation less than 0.05 were determined as differentially expressed proteins. KOG was analyzed using the WebMGA tool [82]. The UniProtKB identifiers of proteins were retrieved from the UniProtKB database [83], and the identified proteins were subjected to GO [84] and KEGG pathway [85] analyses. The fundamental functions of proteins and metabolic pathways were counted and analyzed. The enrichment of differentially regulated proteins in GO terms and metabolic pathways was carried out using the following formula: *m*/*n* > *M*/*N*, where *N* is the number of all proteins with GO or KEGG pathway annotation information; *n* is the number of the differentially regulated proteins with GO or KEGG pathway annotation information; *M* is the number of proteins with a given GO term or KEGG pathway annotation; and *m* is the number of the differentially regulated proteins with a given GO term or KEGG pathway annotation. The GO terms or KEGG pathways with *P* values < 0.05 as threshold in the Chi-square test were considered as enriched GO terms or KEGG pathways by the biofuel-responsive proteins.

### qRT-PCR analysis

The algal cells were collected by centrifugation at 8000×*g* for 5 min at 4 °C. Approximately 10 mg of cell pellets were resuspended in 1 ml TRIzol reagent (Invitrogen) for RNA preparation according to manufacturers’ instructions, and the purified RNA was dissolved in 50 μL DEPC-treated water. Total RNA was quantified, and 1 μg of RNA was digested by RNase-free DNase followed by reverse transcription using a SuperScript VILO master mix for 1st strand cDNA synthesis (Invitrogen). Primers targeting *C. reinhardtii* genes were designed using the Primer Express v3.0 software. The gene name and primer sequences are listed in Additional file 8: Table S7. Real-time PCR analysis was performed on an ABI Vii7™ Real-time PCR System (applied biosystems) using Fast SYBR Green Master Mix kit (applied biosystems). The actin gene was amplified as internal controls. The relative expression level of target genes was calculated by the formula 2^−ΔΔCt^, where $\Delta \Delta C_{\text{t}} = \left( {C_{\text{t, target gene}} - C_{\text{t, actin gene}} } \right)_{\text{stress}} - \left( {C_{\text{t, target gene}} - C_{\text{t, actin gene}} } \right)_{\text{control}} .$

## Additional files

**Additional file 1: Figure S1.** Cell morphology observation under light microscope (100 ×). Scale bars of 50 μm were indicated.

**Additional file 2: Table S1.** Accession numbers of proteins detected in this study.

**Additional file 3: Table S2.** List of differentially regulated proteins in ethanol treatment.

**Additional file 4: Table S3.** List of differentially regulated proteins in butanol treatment.

**Additional file 5: Table S4.** GO annotation of proteins detected in this study.

**Additional file 6: Table S5.** GO enrichment analysis of differentially regulated proteins in ethanol treatment.

**Additional file 7: Table S6.** GO enrichment analysis of differentially regulated proteins in butanol treatment.

**Additional file 8: Table S7.** Primer pairs used in qRT-PCR analysis.

## Abbreviations

GO: gene ontology
Hsps: heat shock proteins
iTRAQ: isobaric tag for relative and absolute quantitation
KOG: eukaryotic cluster of orthologous groups of proteins
LC–MS/MS: liquid chromatography–tandem mass spectrometry
qRT-PCR: real-time quantitative reverse transcription PCR
