# Supplementary figures and images for: Metabolic responses to ethanol and butanol in Chlamydomonas reinhardtii
Source: Biotechnol Biofuels. 2017 Oct 17;10:239. doi: 10.1186/s13068-017-0931-9 (PMC5646117; doi:10.1186/s13068-017-0931-9)

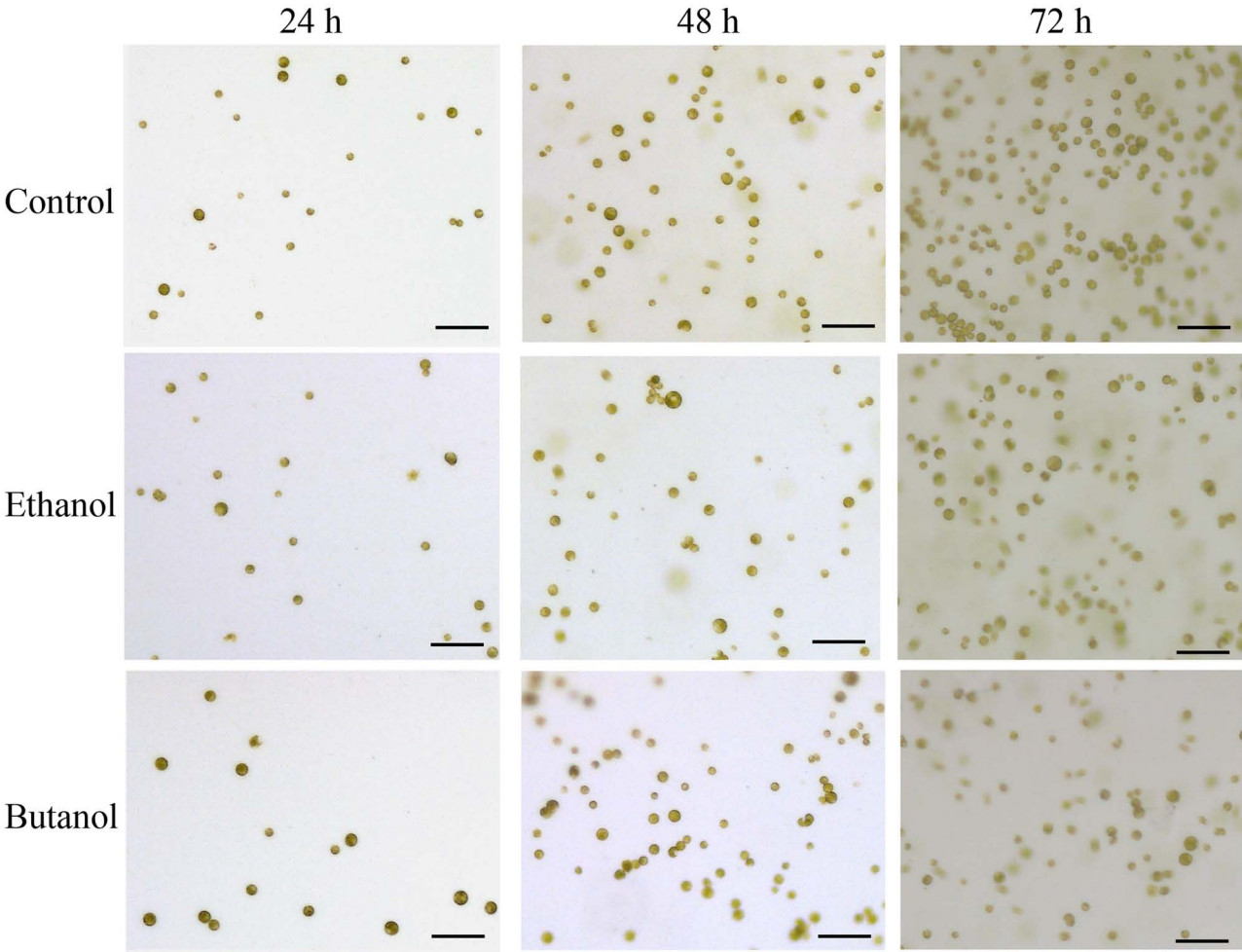

Supplement: Supplementary file 1 — Additional file 1: Figure S1. Cell morphology observation under light microscope (100 ×). Scale bars of 50 μm were indicated. [file 13068_2017_931_MOESM1_ESM.pdf]
